# Synergistic effect of intrathecal fentanyl and bupivacaine in spinal anesthesia for cesarean section

**DOI:** 10.1186/1471-2253-5-5

**Published:** 2005-05-17

**Authors:** Jaishri Bogra, Namita Arora, Pratima Srivastava

**Affiliations:** 1 Deptt. of Anesthesiology, Kings" George Medical University, Lucknow, India; 2 Deptt. of Pharmacokinetics and Metabolism, Central Drug Research Institute, Lucknow, India

## Abstract

**Background:**

Potentiating the effect of intrathecal local anesthetics by addition of intrathecal opiods for intra-abdominal surgeries is known. In this study by addition of fentanyl we tried to minimize the dose of bupivacaine, thereby reducing the side effects caused by higher doses of intrathecal bupivacaine in cesarean section.

**Methods:**

Study was performed on 120 cesarean section parturients divided into six groups, identified as B_8_, B_10 _and B _12.5 _8.10 and 12.5 mg of bupivacaine mg and FB_8_, FB_10 _and FB _12.5 _received a combination of 12.5 μg intrathecal fentanyl respectively. The parameters taken into consideration were visceral pain, hemodynamic stability, intraoperative sedation, intraoperative and postoperative shivering, and postoperative pain.

**Results:**

Onset of sensory block to T6 occurred faster with increasing bupivacaine doses in bupivacaine only groups and bupivacaine -fentanyl combination groups. Alone lower concentrations of bupivacaine could not complete removed the visceral pain. Blood pressure declined with the increasing concentration of Bupivacaine and Fentanyl. Incidence of nausea and shivering reduces significantly whereas, the postoperative pain relief and hemodynamics increased by adding fentanyl. Pruritis, maternal respiratory depression and changes in Apgar score of babies do not occur with fentanyl.

**Conclusion:**

Spinal anesthesia among the neuraxial blocks in obstetric patients needs strict dose calculations because minimal dose changes, complications and side effects arise, providing impetus for this study. Here the synergistic, potentiating effect of fentanyl (an opiod) on bupivacaine (a local anesthetic) in spinal anesthesia for cesarian section is presented, fentanyl is able to reduce the dose of bupivacaine and therefore its harmful effects.

## Background

Spinal anesthesia is the preferred means for cesarean section, being simple to perform, economical and produces rapid onset of anesthesia and complete muscle relaxation. It carries high efficiency, involves less drug doses, minimal neonatal depression, awake mother and lesser incidences of aspiration pneumonitis. However, it also produces a fixed duration of anesthesia, postdural puncture, headache, hypotension and lesser control of block height [1].

Bupivacaine, an amide type of local anesthetic, has high potency, slow onset (5–8 minutes) and long duration of action (1.5–2 hours). For cesarean section intrathecal dose of hyperbaric bupivacaine is 12 to 15 mg [2]. Cesarean delivery requires traction of peritoneum and handling of intraperitoneal organs, resulting in intraoperative visceral pain. With higher doses of hyperbaric bupivacaine, incidence of intraoperative visceral pain associated with higher blocks [4] is reduced [3].

Opiods have been a choice in regional (intrathecal and epidural routes) anesthesia to improve the antinociceptive effect of local anesthetics. Morphine [5], and fentanyl [6], are being used intrathecally, together with local anesthetics in cesarean section.

This study aims to monitor the effect of intrathecal fentanyl + bupivacaine on reduction of higher blocks incidence simultaneously improving the quality and avoiding the complications of higher doses of local anesthetics used in spinal anesthesia in cesarean section. The study can be implicated to select the best possible combination of local anesthetics used in spinal anesthesia in cesarean section.

## Methods

The study was cleared from the Ethics Committee of the University and written consent was taken from patients who participated in this study. All the patients taken for this study belonged to American Society of Anesthesiology (ASA) grade 1 or 2. None of the patients had any contradiction for spinal anesthesia. Complicated pregnancies such as multiple pregnancies, pregnancy induced hypertension and placenta previa were excluded. Also the antenatal patients with acute fetal distress were excluded, keeping the respiratory depressant effect of fentanyl in mind.

Prospective single-blind study was performed on 120 parturients. In the first group of 60 parturients we tried to find the optimal dose of intrathecal bupivacaine, which was not associated with visceral pain. In the second group of 60 parturients intrathecal fentanyl was added to varying dose of bupivacaine. The second group was made with the idea to find out the lowest dose of bupivacaine-fentanyl combination that was not associated with visceral pain. The first group of 60 parturients was further subdivided into 3 subgroups receiving 8,10 or 12.5 mg of 0.5% hyperbaric intrathetal bupivacaine respectively. The second group also of 60 parturients was again subdivided into 3 subgroups receiving 8,10 or 12.5 mg of 0.5% hyperbaric intrathetal bupivacaine mixed with 12.5 μg of intrathecal fentanyl. All parturients were given rapid fluid infusion of 1 to 1.5 litres ringer lactate via 18 gauge venous catheter. Spinal anesthesia was given in lateral position. For lumbar puncture 25 gauge needle was used. Immediately after the block each parturient was placed with 10 cm wedge under right hip. Pulse and noninvasive blood pressure were measured every 5 minutes for the first 30 minutes and thereafter every 10 minutes. If the systolic blood pressure fell below 90 mm HG, additional vasopressor support was given.

Sensory block was tested by pinprick at the left midclavicular line till the block reached T6 when the surgical incision will be allowed. Degree of motor block was assessed by using Bromage scale (BS).

Intraoperative pain was checked whenever the parturients complained of discomfort or pain during operation and expressed as visual analogue scale (VAS 0–1000 mm) by the parturient. Each time VAS exceeded 30–50 μg fentanyl was given IV.

Muscle relaxation was assessed clinically and rated as poor, fair, good or excellent and score of 1,2,3 or 4 was given for each description respectively.

Incidence of nausea, vomiting, itching, shivering pruritus and sedation during operation, the time required for sensory recovery to T10, motor recovery to B0 and the onset of postoperative pain was recorded. All these time variables were measured from the beginning of the spinal injection. Apgar score of all the babies at 1, 5 and 10 minutes and maternal depression were also recorded.

## Results

The study was performed on 120 parturients undergoing caesarian section under spinal anesthesia. Six group of twenty each were made as follows according to the intrathecal medication.

Group I-B_8 _(8 mg 0.5% hyperbaric bupivacaine)

Group II-B_10 _(10 mg 0.5% hyperbaric bupivacaine)

Group III-B _12.5 _(12.5 mg 0.5% hyperbaric bupivacaine)

Group IV-FB_8 _(8 mg 0.5% hyperbaric bupivacaine+12.5 μg fentanyl)

Group V-FB_10 _(10 mg 0.5% hyperbaric bupivacaine +12.5 μg fentanyl)

Group VI-FB _12.5 _(12.5 mg 0.5% hyperbaric bupivacaine +12.5 μg fentanyl)

All six groups were almost of similar age (30 ± 4 years), height (158 ± 6 cm) and weight (54 ± 11 kg).

Onset of sensory block to T6 occurs faster with increasing bupivacaine doses in the alone or < combination groups with fentanyl. Muscle relaxation (degree of muscle relaxation i.e. 4) was excellent in all the patients of all the groups. About 90–100% parturients had complete motor block, with no significant change in different groups. No visceral pain was noticed in any of the combination treated group as well as Group III receiving 12.5 mg of bupivacaine. Incidence of visceral pain was significantly higher in B_8 _and B_10._

Maximum fall in the systolic blood pressure was noticed after 25 minutes in all the groups. Depending upon the % of systolic blood pressure fall the following series can be drawn: FB _12.5_>B _12.5_>FB_10_>B_10_>FB_8_>B_8_. On comparing the hemodynamic stability of equipotent dose of bupivacaine and bupivacaine-fentanyl, we found that the later is more stable. We found the intraoperative hypotension increases with increasing doses of bupivacaine however, alongwith fentanyl it increased more. Bradycardia was found in 10–15% cases in each group. There exists no statistical significance in the incidence of Bradycardia in different groups.

**Figure 1:**
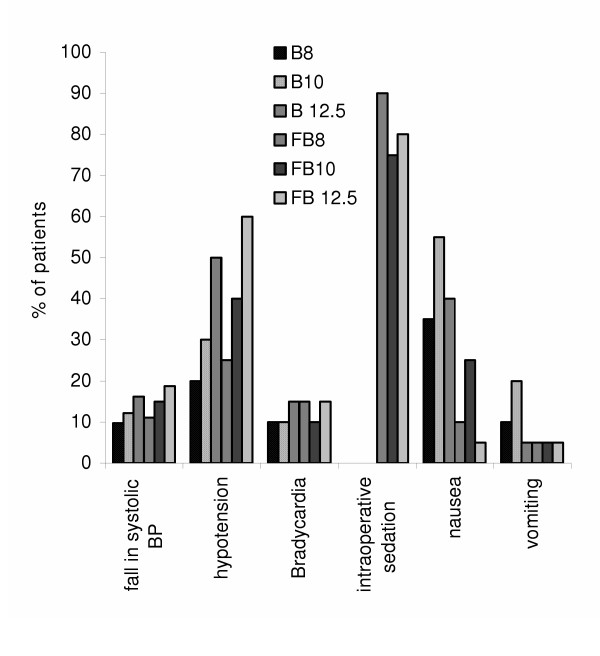
Alterations in different clinical profiles of six groups

**Figure 2:**
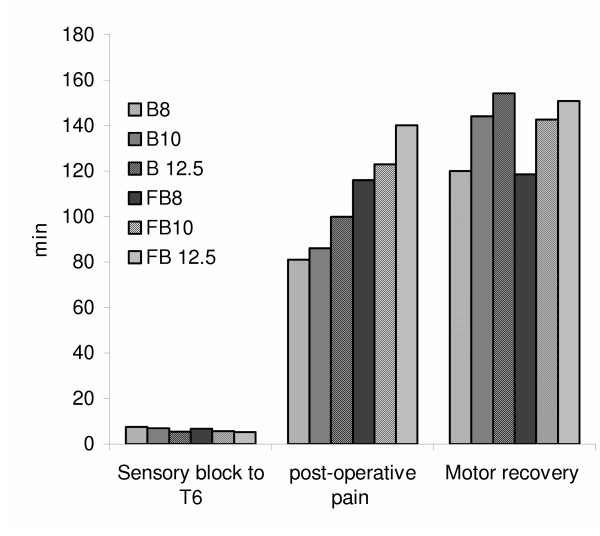
Alteration in different clinical syptoms in 6 groups

There was no intraoperative sedation in bupivacaine whereas 75–90% of parturient of bupivacaine-fentanyl combination groups were drowsy but arousable. Incidence of vomiting was more in bupivacaine alone group than in combination. Duration of post-operative analgesia increased with increasing dose of bupivacaine. Also addition of fentanyl to bupivacaine increased the postoperative analgesic effect. Motor recovery takes longer with increasing doses of bupivacaine alone or in combination with fentanyl. Data of the above-mentioned clinical parameters (if they are significantly different from each other) are given in Fig. 1 and 2.

Apgar score (9.85 ± 0.37 at 10 minutes) of babies was unaffected when additive of 12.5 μg of intrathecal fentanyl was used in cesarean section.

## Discussion

Recent trends of obstetric anesthesia show increased popularity of regional anesthesia amongst obstetric anesthetists. General anesthesia is associated with higher mortality rate in comparison to regional anesthesia. However, regional anesthesia is not without risk. Deaths in regional anesthesia are primarily related to excessive high regional blocks and toxicity of local anesthetics. Reduction in doses and improvement in technique to avoid higher block levels and heightened awareness to the toxicity of local anesthetics have contributed to the reduction of complications related with regional anesthesia [7]. Spinal anesthesia among the neuraxial blocks in obstetric patients needs more strict dose calculations as the drugs are directly injected in intrathecal space. With minimum dose changes, the chances of complications and side effects are enhanced [1]. Above-mentioned factors provided us the impetus to perform this study.

These days 0.5% heavy bupivacaine is used commonly for spinal and epidural anesthesia. It was decided to combine it with intrathecal fentanyl to provide adequate depth of anesthesia with lesser doses of bupivacaine [8]. Fentanyl is a lipophilic opiod and is preferred for having a rapid onset and short duration of action with lesser incidence of respiratory depressions. Our results of onset time of sensory block to T_6 _corroborrate with that of Randalls *et. al *(1991) [9] which state that the onset of sensory block to T_4 _gets faster with increasing bupivacaine doses, whereas, it differs from the observations of Singh *et. al *(19 95) [10].

Complete motor block was achieved in 90–100% of patients with our study; this is in accordance with the results of Pederson *et.al *(1989) [3] and Choi *et.al *(2000) [2]. Visceral pain is a common problem in cesarean section under spinal anesthesia. In our study we found no pain in B _12.5 _group however, visceral pain was not fully abolished with lower doses of bupivacaine. Our results corroborates fully with Choi *et.al *(2000) [2].

It is evident from the results that the depth of anesthesia in FB_8 _group is equivalent to B _12.5 _group. This proves that by adding fentanyl adequate depth of spinal anesthesia can be achieved at much lower doses of bupivacaine. Incidence of hypotension as well as fall in the systolic BP increases with the dose of bupivacaine. However, no significant difference was noticed in the fentanyl added group when compared with their nonadded counterpart.

Bradycardia results from the blockade of sympathetic cardio accelerator fibers and decreased venous return to the heart. In our study bradycardia occurrence was overall 7% with no significant intergroup variation. This is in accordance with Singh et.al (1991) [10]. About 75–90% of the patients become drowsy but arousable with the intrathecal fentanyl addition as compared to those without fentanyl addition. However, there is report where fentanyl addition do not cause any change in the sedation [10], however, similar to the findings of Randalls *et. al *(1991) [10] we found significant reduction in the incidence of nausea by the addition of fentanyl to bupivacaine. Further, negligible incidences of pruritus, shivering or respiratory depression were observed. Also, the apgar score of the babies remained same in all the groups. There was longer duration of postoperative analgesia in fentanyl-bupivacaine groups; this also increases with the increasing dose of bupivacaine. However, motor recovery was not affected by the addition of fentanyl.

## Conclusion

The above study can be concluded as bupivacaine -fentanyl combination leads to abolishment of the visceral pain, reduction in nausea incidence increased hemodynamic stability and increases the duration of post-operative analgesia; however no effect can be seen on bradycardia, nausea, vomiting, shivering, maternal or neonatal respiration. Thus, overall the combined effect of fentanyl-bupivacaine is superior over just bupivacaine alone as fentanyl apart from positive effects, retards the negativity as well as reduces the doses of bupivacine too.

## Competing interests

The author(s) declare that they have no competing interests.

## Authors' contributions

JB is guide and with NA helped in execution of work and PS helped in experiment design and manuscript preparation. All authors read and approved the final manuscript. The authors do not have any financial or non-finanacial competing interests.

## Pre-publication history

The pre-publication history for this paper can be accessed here:


